# Genetic Deletion of *Rheb1* in the Brain Reduces Food Intake and Causes Hypoglycemia with Altered Peripheral Metabolism

**DOI:** 10.3390/ijms15011499

**Published:** 2014-01-21

**Authors:** Wanchun Yang, Wanxiang Jiang, Liping Luo, Jicheng Bu, Dejiang Pang, Jing Wei, Chongyangzi Du, Xiaoqiang Xia, Yiyuan Cui, Shuang Liu, Qing Mao, Mina Chen

**Affiliations:** 1 Laboratory of Molecular Neurobiology, State Key Laboratory of Biotherapy/West China Hospital, Sichuan University, Chengdu 610041, China; E-Mails: yangwanchunscu@gmail.com (W.Y.); wanxjiang@gmail.com (W.J.); lipingluo1987@gmail.com (L.L.); jichengbu@gmail.com (J.B.); weijing3131@gmail.com (J.W.); duchongyangzi@gmail.com (C.D.); xiaoqiangxia@gmail.com (X.X.); cuiyy307@gmail.com (Y.C.); 2 West China School of Preclinical and Forensic Medicine, Sichuan University, Chengdu 610041, China; E-Mail: pangdejiang@gmail.com; 3 Department of Neurosurgery, West China Hospital, Sichuan University, Chengdu 610041, China; E-Mail: liushuang3302@163.com

**Keywords:** *Rheb1*, genetic deletion, food intake, hypoglycemia, ketogenesis

## Abstract

Excessive food/energy intake is linked to obesity and metabolic disorders, such as diabetes. The hypothalamus in the brain plays a critical role in the control of food intake and peripheral metabolism. The signaling pathways in hypothalamic neurons that regulate food intake and peripheral metabolism need to be better understood for developing pharmacological interventions to manage eating behavior and obesity. Mammalian target of rapamycin (*mTOR*), a serine/threonine kinase, is a master regulator of cellular metabolism in different cell types. Pharmacological manipulations of *mTOR* complex 1 (*mTORC1*) activity in hypothalamic neurons alter food intake and body weight. Our previous study identified *Rheb1* (Ras homolog enriched in brain 1) as an essential activator of *mTORC1* activity in the brain. Here we examine whether central *Rheb1* regulates food intake and peripheral metabolism through *mTORC1* signaling. We find that genetic deletion of *Rheb1* in the brain causes a reduction in *mTORC1* activity and impairs normal food intake. As a result, *Rheb1* knockout mice exhibit hypoglycemia and increased lipid mobilization in adipose tissue and ketogenesis in the liver. Our work highlights the importance of central *Rheb1* signaling in euglycemia and energy homeostasis in animals.

## 1. Introduction

Energy homeostasis is a fundamental mechanism used by multicellular organisms to maintain their survival and body growth [1]. It is tightly linked to food intake [2,3]. How the link between food intake and energy homeostasis is controlled remains incompletely understood. Recent studies show that central mammalian target of rapamycin (*mTOR*) signaling plays a critical role in the control of food intake and energy homeostasis [4–6]. *mTOR*, a serine/threonine kinase, is a master regulator of cellular metabolism, including protein and lipid synthesis, and energy production [7–9]. *mTOR* kinase functions in two distinct protein complexes—*mTORC1* (mammalian target of rapamycin complex 1) and *mTOR*C2 (mammalian target of rapamycin complex 2) [10,11]. *mTORC1* activity is activated by growth factors (e.g., insulin) and nutrients (e.g., amino acids) [12], and negatively regulated by GTPase activity of the *TSC* (tuberous sclerosis complex) protein complex encoded by the *TSC1* and *TSC2* genes [13]. In 2006, a landmark paper showed that altered *mTORC1* activity in hypothalamic neurons alters food intake and energy metabolism [4]. Intracerebroventricular injection of leucine in the vicinity of the arcuate (ARC) neurons in hypothalamus activates *mTORC1* activity and reduces food intake. Conversely, injection of *mTORC1* inhibitor rapamycin increases food intake and blunts leptin-mediated anorectic effect [4]. Subsequently, S6 kinase (*S6K*), a downstream substrate of *mTORC1*, is reported to regulate food intake and energy homeostasis [14]. The injection of adenovirus expressing dominant negative (DN) *S6K* in the mediobasal hypothalamus increases food intake, while expressing constitutively active (CA) *S6K* decreases food intake [14]. These studies establish the central control of food intake and energy homeostasis through hypothalamic neurons. However, a recent study shows that genetic deletion of *TSC1* in hypothalamic POMC (proopiomelanocortin) neurons increases *mTORC1* activity, and food intake by *TSC1* knockout mice is increased and associated with increased body weight. Also, intracerebral rapamycin infusion was shown to reduce food intake and body weight in aged mice [15]. These results contradict earlier reports showing that increased *mTORC1* activity in hypothalamic neurons reduces food intake. Although these studies have demonstrated a critical role of *mTORC1* activity in the control of food intake and energy metabolism, most of them are based on pharmacological or viral mediated manipulations of *mTORC1* activity *in vivo*. Therefore, the role of the *mTOR* signaling pathway in the control of food intake and energy metabolism needs further clarification in genetic models. Our previous study shows that *Rheb1* (Ras homolog enriched in brain 1) is required for the activation of *mTORC1* activity in the brain [16]. The genetic deletion of *Rheb1* in neural stem/progenitor cells of the brain (*Rheb1* f/f Nestin-cre, hereafter *Rheb1* ko) renders the *mTORC1* activity undetectable as shown by the absence of phosphorylation of *S6*, whereas *mTORC2* activity is not impaired [16]. The *Rheb1* ko mice show impaired postnatal growth and reduced body weight by 64.7% at 3-week-old, living for about 6 weeks [16]. The reason for the premature death is unknown. Now we show that the *Rheb1* ko mouse reduces food intake and develops severe hypoglycemia and peripheral metabolic adaptations to meet the energy demands of the brain.

## 2. Results and Discussion

### 2.1. Genetic Deletion of *Rheb1* in the Brain Disrupts Food Intake

**Figure 1.**
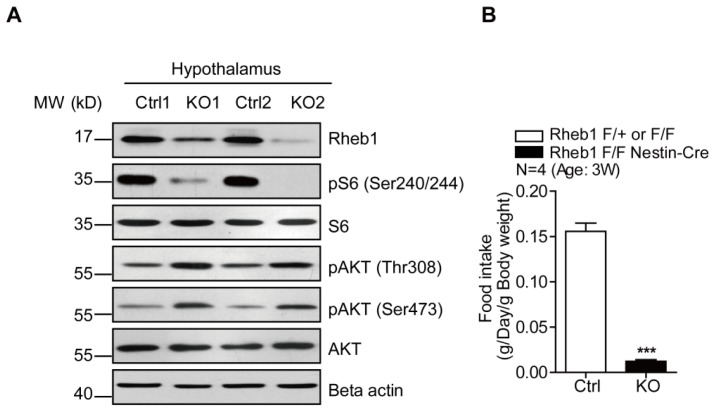
Genetic deletion of *Rheb1* in the brain disrupts food intake. (**A**) Western blotting showing that *Rheb1* and pS6 (Ser240/244) protein levels are reduced in *Rheb1* ko hypothalamus; (**B**) Histograms showing the drastic reduction of food intake in *Rheb1* ko mice compared to controls (*N* = 4). Results are averages of four independent animals. Data represent mean ± SEM. *** *p* < 0.001.

To examine the role of *Rheb1*/*mTORC1* signaling in the regulation of food intake and energy homeostasis, we examined the *mTORC1* activity in *Rheb1* ko mice (*Rheb1* f/f, Nestin-cre) in which *Rheb1* was deleted by *Cre* activity expressed by *Nestin* promoter [17]. We focused on the hypothalamus, because it is critical in the central control of food intake. The results revealed that *mTORC1* activity was dramatically reduced in the hypothalamus of *Rheb1* ko, as indicated by pS6 (Ser240/244) (Figure 1A). This finding is consistent with the effect of *Rheb1* ko on *mTORC1* activity in other brain regions [16]. Given the importance of *mTORC1* activity in hypothalamic neurons, particularly in POMC neurons, in the control of food intake, we examined the food intake of 3-week-old *Rheb1* ko mice. The results showed that the food intake by *Rheb1* ko mice was significantly reduced relative to the control mice (floxed *Rheb1* mice without *Cre*) (*N* = 4) (Figure 1B). These data indicate that central *Rheb1* signaling plays a role in the control of food intake.

### 2.2. Genetic Deletion of *Rheb1* in the Brain Leads to Hypoglycemia

**Figure 2.**
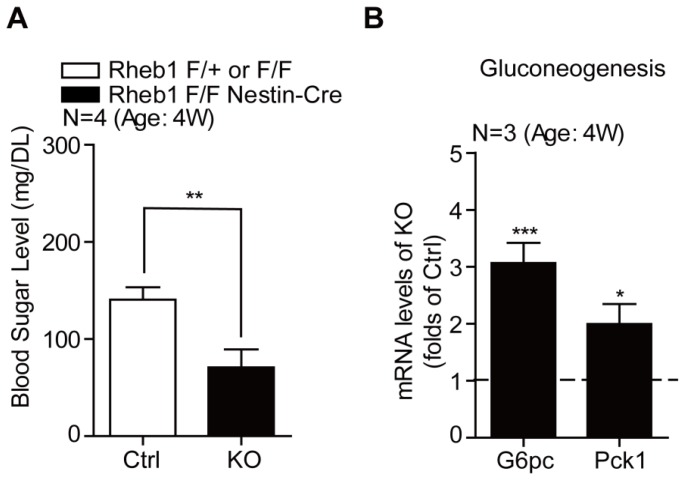
Genetic deletion of *Rheb1* in the brain leads to hypoglycemia. (**A**) Biochemical assays showing the robust reduction of blood glucose level in *Rheb1* ko mice compared controls (*N* = 4). Results are averages of four independent animals. Data represent mean ± SEM. ** *p* < 0.01; (**B**) Real-time PCR assays showing the increasing mRNA levels of *G6pc* and *Pck1* in livers of *Rheb1* ko mice (*N* = 3). Results are averages of three independent animals. Data represent mean ± SEM. * *p* < 0.05, *** *p* < 0.001.

Food intake is critical to maintain blood glucose level and energy homeostasis. We wondered if blood glucose level was altered in *Rheb1* ko mice. Blood glucose measurement shows that *Rheb1* ko mice had a much lower glucose level (70 mg/DL) than that in their control littermates (150 mg/DL) (*N* = 4) (Figure 2A). To further examine the effect of *Rheb1* ko on energy homeostasis, we examined the gluconeogenesis in the liver where *Rheb1*/*mTORC1* activity in hepatocytes was slightly increased (see below). Real-time PCR analysis revealed that the mRNA levels of two canonical gluconeogenesis genes, *G6pc* and *Pck1* [18], were increased in the liver of *Rheb1* ko mice in comparison with those in control livers (Figure 2B). This result suggested that gluconeogenesis was activated in *Rheb1* ko mice to synthesize glucose from non-carbohydrate precursors as part of homeostatic adaptation.

### 2.3. Genetic Deletion of *Rheb1* in the Brain Induces Ketogenesis in the Liver

**Figure 3.**
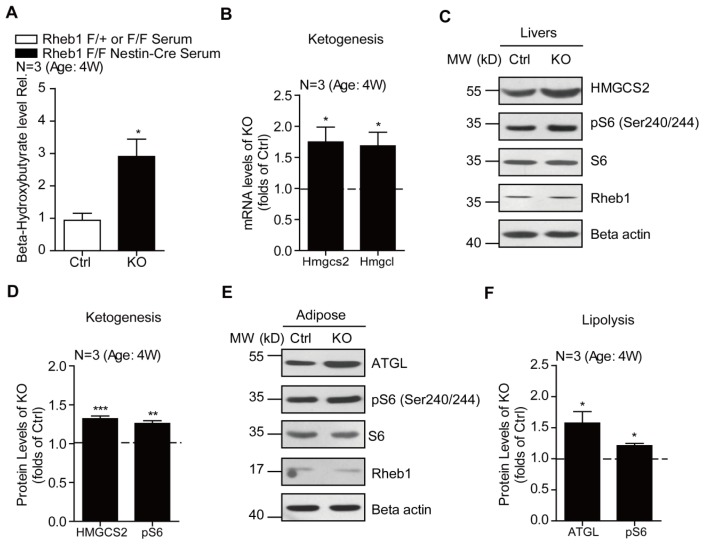
Genetic deletion of *Rheb1* in the brain induces ketogenesis in the liver. (**A**) Biochemical assays showing increased blood ketone levels in 4-week-old *Rheb1* ko mice (*N* = 3). Results are averages of three independent animals. Data represent mean ± SEM. * *p* < 0.05; (**B**) Real-time PCR results showing the increasing levels of ketogenesis genes *Hmgcs2* and *Hmgcl* in livers of *Rheb1* ko mice (*N* = 3). Results are averages of three independent animals. Data represent mean ± SEM. * *p* < 0.05; (**C**,**D**) Western blots and histograms showing the increasing of *HMGCS2* protein level in livers of *Rheb1* ko mice (*N* = 3). Results are averages of three independent animals. Data represent mean ± SEM. ** *p* < 0.01, *** *p* < 0.001; (**E**,**F**) Western blots and histograms showing the increasing of *ATGL* protein level in adipose of *Rheb1* ko mice (*N* = 3). Results are averages of three independent animals. Data represent mean ± SEM. * *p* < 0.05.

To further examine the adaptive metabolic changes as a result of altered *Rheb1*/*mTORC1* activity in the brain, we assayed the ketogenesis in the liver of *Rheb1* ko mice. Ketogenesis is an essential component of metabolic adaptations to meet the energy demand of the brain. We examined ketogenesis in the liver of *Rheb1* ko mice and found that the ketogenesis was increased compared to the controls. This was demonstrated through increased levels of beta-hydroxybutyrate in serum of *Rheb1* mice (*N* = 3) (Figure 3A) and increased mRNA levels of hydroxy-methyl-glutaryl coenzyme A synthase 2 (HMG-CoA synthase 2, *Hmgcs2*) and HMG-CoA lyase (*Hmgcl*) (*N* = 3)—enzymes that are critical for ketogenesis in the liver [19,20] (*N* = 3) (Figure 3B). Consistent with the mRNA increase of *Hmgcs2*, its protein level was also increased by 32.1% (Figure 3C,D). Increased ketogenesis is normally linked to increased lipid mobilization to provide free fatty acids for biosynthesis of ketone bodies in the liver. The majority of free fatty acids are generated as a consequence of the breakdown of triglycerides in adipose tissue and transferred to the liver through circulation for ketogenesis [21]. Therefore, we assayed the protein level of adipose triglyceride lipase (*Atgl*) as an indicator of lipolysis. Western blotting revealed a significant increase of *ATGL* protein level in the adipose tissue of *Rheb1* ko mice as compared to the controls (*N* = 3), suggesting increased lipid mobilization in *Rheb1* ko mice. Collectively, our data supports the notion that genetic deletion of *Rheb1* in the brain would increase adaptive lipid mobilization and ketogenesis to meet the energy demand of the brain.

### 2.4. Genetic Deletion of *Rheb1* in the Brain Increases Liver Lipid Droplets and Beta Oxidation

**Figure 4.**
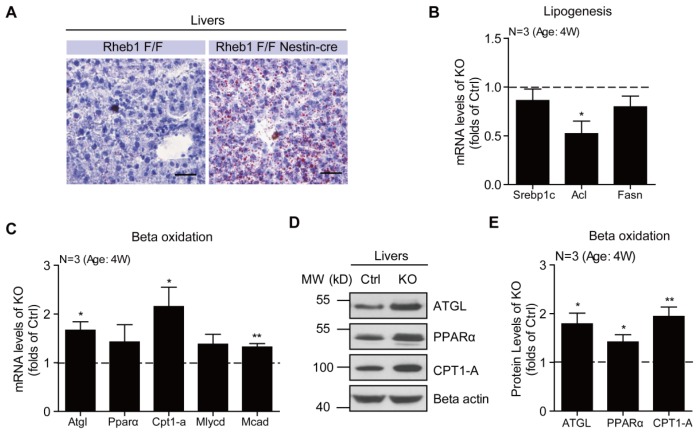
Genetic deletion of *Rheb1* in the brain increases liver lipid droplets and beta oxidation. (**A**) Representative oil red O staining images showing increased number of lipid droplets in livers of *Rheb1* ko mice. Bar 25 μm; (**B**) Real-time PCR assays showing that hepatic lipogenesis genes *Srebp1c*, *Acl* and *Fasn* are not increased in livers of *Rheb1* ko mice (*N* = 3). Results are averages of three independent animals. Data represent mean ± SEM. * *p* < 0.05; (**C**) Real-time PCR assays showing the increasing of mRNA levels of beta-oxidation genes, *Atgl*, *Pparα*, *Cpt1-a*, *Mlycd* and *Mcad* in livers of *Rheb1* ko mice (*N* = 3). Results are averages of three independent animals. Data represent mean ± SEM. * *p* < 0.05, ** *p* < 0.01; (**D**,**E**) Western blots and histograms showing the increasing of protein levels of *ATGL*, *PPARα* and *CPT1-A* in livers of *Rheb1* ko mice (*N* = 3). Results are averages of three independent animals. Data represent mean ± SEM. * *p* < 0.05, ** *p* < 0.01.

The excessive influx of free fatty acids to the liver as a result of increased lipid mobilization tends to increase the formation of lipid droplets in the liver [22]. In support of this notion, we found that the lipid accumulation in the form of lipid droplets was significantly increased in the liver of *Rheb1* ko mice. By oil red O staining, we found a significant increase of lipid droplets in the liver of 4-week-old *Rheb1* ko mice relative to controls (Figure 4A). Moreover, real-time PCR results showed that the mRNA levels of genes that were critical for lipogenesis, such as *Srebp1c* [23], *Acl* [24] and *Fasn* [23,24], were not increased in the liver of *Rheb1* ko mice (*N* = 3) (Figure 4B), suggesting that this lipid accumulation was not due to increased *de novo* lipogenesis in the liver. The fatty acids derived from adipose tissue are utilized by beta-oxidation to generate acetyl-CoA for energy production [25,26]. To investigate whether the beta-oxidation was activated to oxidize the excessive fatty acids in the liver of *Rheb1* ko mice, we assayed the mRNA and protein levels of genes that are critical for beta-oxidation. The results showed that the mRNA levels of *Atgl* [27], *Pparα* [28], *Cpt1-a* [29], *Mlycd* [30] and *Mcad* [31], were all increased in the liver of *Rheb1* ko mice relative to controls (*N* = 3) (Figure 4C). Consistent with the increases in mRNA levels, protein levels for *ATGL*, *PPARα* and *CPT1-A* were also increased as shown by Western blotting (*N* = 3) (Figure 4D,E). All these results support the notion that adipose-derived lipids are transferred to the liver for beta-oxidation and subsequent ketogenesis to meet the energy demand of the brain in *Rheb1* ko mice.

The present study uses genetic approaches to demonstrate a significant role of central *Rheb1* in the control of food intake and energy homeostasis. Because *mTORC1* activity in hypothalamic neurons is critical for food intake [4,15] and genetic deletion of *Rheb1* inhibits *mTORC1* activity in hypothalamus (Figure 1A), it is probable that the reduced food intake and hypoglycemia in *Rheb1* ko mice is due to reduced *mTORC1* activity in hypothalamic neurons. To meet the energy demand of the brain, *Rheb1* ko mice develop adaptive changes in peripheral systems, such as lipid mobilization (Figure 3E), ketogenesis (Figure 3A) and gluconeogenesis (Figure 2B).

We noted that the increase in ketogenesis in the liver of *Rheb1* ko mice is not due to loss of *Rheb1* activity in hepatic cells, because we did not detect any significant change to *Rheb1* protein level in livers of *Rheb1* ko mice (Figure 3C). This non-cell autonomous effect of *Rheb1* is consistent with the report that *Nestin* is expressed in only hepatic stellate cells that account for only 5% of the total number of cells in the mouse liver [32]. Moreover, we found that *mTORC1* activity was slightly increased in hepatic and adipose tissues of *Rheb1* ko mice (Figure 3C,E), suggesting that the increased ketogenesis and lipid mobilization in *Rheb1* ko mice is a secondary effect of loss of central *Rheb1* activity.

Animals develop multiple cellular mechanisms to control food intake to maintain energy homeostasis [1]. How food intake is regulated at the molecular level remains an unresolved question. Recent progress has focused on the importance of hypothalamic neurons in the control of food intake. As a central regulator of metabolisms, *mTOR* not only controls the lipid and protein biosynthesis, but also manipulates the feeding behaviors. Several studies have showed that *mTOR* signaling may serve as a negative regulator of food intake. For example, activation of hypothalamic *mTORC1* signaling by central administration of leucine decreases food intake and body weight, and inhibition of *mTORC1* signaling by rapamycin injection blunts leptin’s anorectic effect [4]. This effect seems to be mediated by *S6K*, because *S6K*, which is a major downstream factor of *mTORC1*, also negatively regulates food intake [14]. However, contradictive evidence points out that activation of *mTORC1* by *TSC* ablation in hypothalamic POMC neurons increases food intake and body weight, while intracerebral rapamycin infusion restores it [15]. These controversial results raise the questions of how the *mTOR* pathway controls hypothalamic food intake center, anorexigenic or orexigenic. Of note, most of these studies are based on pharmacological or viral-mediated manipulations of *mTORC1* activity *in vivo*. Therefore, studies of genetically modified mouse models are necessary to clarify the role of *mTOR* signaling in the control of food intake and energy homeostasis. Here, we examined the effect of *Rheb1* deletion in the brain on food intake and subsequent alterations to peripheral metabolism. Our results demonstrate that as a result of *Rheb1* deletion, *mTORC1* activity in the hypothalamus is significantly reduced and the *Rheb1* ko mice exhibit significantly reduced food intake and hypoglycemia. To meet the energy demand of the brain, lipid mobilization is activated to increase the biosynthesis of ketone bodies in the liver.

The central regulation of food intake is an immensely complex process involving the coordinated activities of multiple cerebral loci, such as the limbic system [33] and the cerebral cortex [34]. The neuronal processes from these loci project onto hypothalamic neurons and regulate appetite. Recent imaging studies indicate the awareness of hunger involves cortical functions [35]. Since the *mTORC1* activity in the cortex is also affected in *Rheb1* ko mice, presumably, the awareness of hunger is also altered by *Rheb1* deletion. Although *Rheb1* has been widely accepted as a key activator of *mTORC1* signaling *in vitro* and *in vivo*, there may be additional *mTORC1*-independent effects of *Rheb1*. Because genetic deletion of *Rheb1* in the brain increases *AKT* activity by the *mTORC1*-*S6K1*-*IRS* feedback pathway, future studies will address whether increased *AKT* activity contributes to the reduced food intake in *Rheb1* ko mice. Again, genetic deletion of *mTOR* kinase or *raptor* may also help to further clarify whether the effect of *Rheb1* on food intake is mediated by *mTORC1* signaling.

## 3. Experimental Section

### 3.1. Reagents and Antibodies

The biochemical kit of beta-hydroxybutyrate was purchased from Biovision (Milpitas, CA, USA). The following antibodies, *ATGL* and pS6 (S240/244), were from Cell Signaling Technology (Danvers, MA, USA). Anti-β-actin was purchased from Millipore (Billerica, MA, USA). And *CPT1-A* antibody was from Abcam (Cambridge, UK). *PPARα* was from Thermo Scientific (Rockford, IL, USA), and *HMGCS2* from LifeSpan BioScience (Seattle, WA, USA). Anti-*Rheb1* antibody has been described previously [16].

### 3.2. Animals

*Rheb1* ko mice were generated by breeding floxed *Rheb1* mice with *Nestin-cre* deleter mice, and genotyped as previously described [16]. Both the *Rheb1* ko and control littermates were housed in environmentally controlled conditions with a 12-hour light/dark cycle, and fed normal chow. All mouse work was done in accordance with the Animal Care and Use Committee guidelines of Sichuan University West-China Hospital.

### 3.3. Blood Glucose Measurement

Blood glucose levels were measured in a drop of blood obtained from tails of 4-week-old *Rheb1* ko mice and control littermates at 10:00 a.m. in the fed state. The blood glucose analysis was performed using the Roche Accu-Chek Active System (Roche Diagnostics, Mannheim, Germany).

### 3.4. Western Blotting

Mouse adipose, liver and hypothalamus extracts were made with lysis buffer (2% SDS with protease and phosphatase inhibitors). Same amounts of proteins were loaded into SDS-PAGE gels and blotted with various antibodies, according to standard Western blotting procedures.

### 3.5. RNA Extraction and Real-Time PCR Assay

Total RNAs were extracted from tissues using Trizol reagent (Invitrogen, Carlsbad, CA, USA). RNA was subjected to reverse transcription with reverse transcriptase according to the manufacturer’s instructions (Fermentas, Glen Burnie, MD, USA). Quantitative real-time PCR was performed using the Bio-Rad iQ5 system (Bio-Rad, Hercules, CA, USA), and the relative gene expression was normalized to internal control as beta-actin. Primer sequences for SYBR Green probes of target genes were as follows:

*G6pc*: 5′-GTGTCCGTGATCGCAGACC-3′ and 5′-GACGAGGTTGAGCCAGTCTC-3′;
*Pck1*: 5′-T TGAGAAAGCGTTCAATGCCA-3′ and 5′-CACGTAGGGTGAATCCGTCAG-3′;
*Hmgcs2*: 5′-ATA CCACCAACGCCTGTTATGG-3′ and 5′-CAATGTCACCACAGACCACCAG-3′;
*Hmgcl*: 5′-ACTA CCCAGTCCTGACTCCAA-3′ and 5′-TAGAGCAGTTCGCGTTCTTCC-3′;
*Srebp1c*: 5′-GGAGCC ATGGATTGCACATT-3′ and 5′-GCTTCCAGAGAGGAGGCCAG-3′;
*Acl*: 5′-GAAGCTGACCTT GCTGAACC-3′ and 5′-CTGCCTCCAATGATGAGGAT-3′;
*Fasn*: 5′-TGGGTTCTAGCCAGCAGA GT-3′ and 5′-ACCACCAGAGACCGTTATGC-3′;
*Atgl*: 5′-TGTGGCCTCATTCCTCCTAC-3′ and 5′-TGCTGGATGTTGGTGGAGCT-3′;
*Pparα*: 5′-TGTTTGTGGCTGCTATAATTTGC-3′ and 5′-GCAACTTCTCAATGTAGCCTATGTTT-3′;
*Cpt1-a*: 5′-GGAGAGAATTTCATCCACTTCCA-3′ and 5′-CTTCCCAAAGCGGTGTGAGT-3′;
*Mlycd*: 5′-GCACGTCCGGGAAATG AAC-3′ and 5′-GCCTCACACTCGCTGATCTT-3′;
*Mcad*: 5′-TTTCGAAGACGTCAGAGTGC-3′ and 5′-TGCGACTGTAGGTCTGGTTC-3′;
*Beta-actin*: 5′-GAGACCTTCAACACCCCAGC-3′ and 5′-ATGTCACGCACGATTTCCC-3′.

### 3.6. β-Hydroxybutyrate Assay

Serum samples from *Rheb1* ko or control mice (fed normally) were collected into a 96-well plate by retroorbital bleeding according to standard procedure. The procedure was performed at approximately same time on the days when the blood glucose assay was carried out. Beta-hydroxybutyrate levels were measured using a commercial diagnostic kit of Biovision (Catalog. #K632-100, Milpitas, CA, USA) according to the manufacturer’s instruction.

### 3.7. Oil Red O Staining

Standard Oil Red O staining was performed on frozen liver sections to evaluate tissue lipid content. After sacrifice, fresh livers were frozen immediately and subsequently cut into 10 μm sections by Cryostat Microtome. The staining solution was prepared by dissolving 0.5 g oil-red-O (Sigma, St. Louis, MO, USA) in 100 mL of isopropanol; 60 mL of this solution was mixed with 40 mL of distilled water. After 1 h at room temperature the staining solution was filtered and added to liver slices for 15 min. The staining solution was then removed and liver slices were washed twice with distilled water.

### 3.8. Food Intake Assay

For food intake experiments, three-week-old *Rheb1* ko mice and control littermates were separated and each mouse was raised with chow (10 g/day) in a single cage. After 24 h, all the remainder food was collected and quantified. The experiments lasted for five days. Then the mass of intake food was normalized to mouse body weight for statistical analysis.

### 3.9. Statistical Analysis

All results of Western blotting, real-time PCR, and food intake across time were presented as mean ± SEM from a minimum of three or four independent experiments. Data from Western-blots were analyzed by ImageJ software (U.S. National Institutes of Health (NIH), Bethesda, MD, USA). *P* values were calculated using the Student’s *t* test for normally distributed data, and the value 0.05 (*), 0.01 (**) and 0.001 (***) was assumed as the level of significance for the statistic tests carried out.

## 4. Conclusions

In conclusion, our work highlights an important role of central *Rheb1* in euglycemia and energy homeostasis in animals, which is mostly likely mediated by *mTORC1* signaling. Our study sets the stage to examine how *Rheb1*/*mTORC1* activity in hypothalamic neurons controls food intake and alters peripheral metabolism.

## Abbreviations

*mTOR*: mammalian target of rapamycin
*mTORC1*: mammalian target of rapamycin complex 1
*mTORC2*: mammalian target of rapamycin complex 2
*Rheb1*: Ras homolog enriched in brain 1
ARC: arcuate
POMC: proopiomelanocortin
DN: dominant negative
CA: constitutively active
*Rheb1* ko: *Rheb1* f/f Nestin-cre
*HMG-CoA*: hydroxy-methyl-glutaryl coenzyme A
*HMGCS2*: HMG-CoA synthase 2
*HMGCL*: HMG-CoA lyase
*ATGL*: adipose triglyceride lipase
